# The allium stent for the complex ureteral stricture‐deeper experience of a series case review

**DOI:** 10.1002/bco2.70042

**Published:** 2025-07-01

**Authors:** Hanqi Lei, Yajiao Cui, Mengjun Huang, Donggen Jiang, Yamei Li, Jun Pang

**Affiliations:** ^1^ Department of Urology, Kidney and Urology Center, Pelvic Floor Disorders Center, The Seventh Affiliated Hospital Sun Yat‐sen University Shenzhen China

**Keywords:** allium stents, cases review, deeper experience, new findings, ureteral stricture

## Abstract

**Background:**

Allium stents are widely used in patients with ureteral stricture, with ongoing research continuously evaluating their clinical safety and efficacy.

**Objective:**

This study aimed to describe our technique and report the outcomes of Allium stent in the treatment of refractory ureteral strictures.

**Design, setting and participants:**

We retrospectively collected perioperative data on all patients treated with Allium stents in our department between January 2017 and April 2024 and assessed their clinical outcomes.

**Surgical procedure:**

Following ureteroscopy, a guidewire was advanced under fluoroscopic guidance into the renal pelvis. The retrograde ureterography was performed to determine the location and length of the ureteral stricture. Dilation was performed using a ureteral balloon dilator, a flexible ureteroscope sheath, or a rigid ureteroscope. Subsequently, the Allium stent was deployed into the stricture segment and confirmed via fluoroscopic imaging.

**Results:**

A total of 23 patients (25 ureters) were included, with a mean age of 57.7 years (32–76 years). The mean length of ureteral strictures was 4.5 cm (range: 1–18 cm). All stents were successfully positioned. As of December 2024, the stent patency rate was 68%, with a median follow‐up of 39.5 months (13–67 months). In eight patency failure cases, the mean indwelling time was 14 months, with the shortest recorded duration being 2 months. Causes of failure included four (50%) stent migration, one (12.5%) encrustation, two (25%) persistent stenosis and severe infection (12.5%). Management strategies for these cases included two (25%) stent removal, two (25%) robot‐assisted pyeloureteroplasty, one (12.5%) ureterolithotripsy, one (12.5%) exchange with a new Allium stent, one (12.5%) add new Allium stent, and one (12.5%) replacement with a different type of metal stent. Notably, one case of a ruptured ureter was successfully bridged with an Allium stent, and another case of a uretero‐vaginal fistula was effectively treated with Allium stent, both without complications.

**Conclusions:**

Allium stents appear to be a feasible and effective treatment for various ureteral strictures, including cases of ureteral perforation and rupture. However, long‐term complications such as stent migration and occlusion remain challenges that should not be overlooked.

## 1 INTRODUCTION

Ureteral stenosis is a common condition of the urinary system, characterized by the narrowing of the ureteral lumen due to various causes, including iatrogenic injury and radiation treatment.^1^, ^2^, ^3^, ^4^, ^5^ If left untreated, prolonged obstruction and narrowing of the ureter can lead to hydronephrosis, decreased renal function, renal failure, and even the loss of a renal unit.^6^, ^7^ Iatrogenic ureteral strictures are more likely to occur in the lower third of the ureter compared to the middle and upper thirds.^8^, ^9^

To prevent renal dysfunction, ureteral strictures can be managed through various approaches, including open surgery, laparoscopic or robotic reconstruction, endoscopic ureterotomy, nephrostomy and stent insertion.^1^, ^2^ However, for patients who develop ureteral stricture following surgery and/or radiation treatment for malignancy, performing open surgery can be challenging due to the formation of intra‐abdominal adhesions.^10^, ^11^, ^12^ Thus, managing complex ureteral strictures remains a significant challenge in clinical practice.

The insertion of a nephrostomy tube is often a direct, effective and relatively simple solution for patients with ureteral stenosis and hydronephrosis; however, it is typically uncomfortable for patients.^13^, ^14^ An alternative option for patients who are unwilling or unfit for surgical intervention is the use of a continuously indwelling ureteral stent with periodic exchanges. Double‐J stents are commonly used in these cases, but they come with side effects, including infection, lumbar pain, haematuria, bladder irritation and tube encrustation, which can limit patients' ability to engage in strenuous physical activities.^13^ Additionally, double‐J stents have a short retention time, typically requiring replacement every 3–6 months. This can lead to increased treatment costs and frequent hospital admissions.

In contrast, Allium stents, which are self‐expanding covered metal stents, offer several advantages: reduced tissue ingrowth, large‐calibre, high anti‐compression force and the ability to remain safely in place for up to 3 years without the need for exchange.^15^ These attributes make Allium stents a promising option for treating ureteral stenosis in current clinical practice.^16^, ^17^, ^18^, ^19^, ^20^ The goal of this study was to report the long‐term outcomes of all patients who were treated with the Allium stent in our department.

### 1.1 Methods

A retrospective study was conducted on patients with ureteral stenosis who were treated with Allium stent (Allium Medical Solutions Ltd., Caesarea, Israel) between January 2017 and April 2024 at the Department of Urology, The Seventh Affiliated Hospital, Sun Yat‐sen University, Shenzhen, China. All participants were informed of the risks and benefits of stent placement prior to surgery. Stent patency was defined as the presence of the stent in situ without migration, unplanned stent exchange or recurrent ureteral obstruction, as indicated by increased hydronephrosis and associated renal function deterioration. A kidney, ureter and bladder (KUB) X‐ray was performed within 1 week after stent insertion to confirm the correct position of the stent. Patients were advised to undergo CT or KUB 1 month after insertion. Follow‐up included regular medical imagings, serum creatinine measurements, urine cultures, urinalysis and serum biochemistry tests every 3 months.

### 1.2 Allium stent

The Allium stent is designed for the treatment of ureteral strictures. It is a self‐expanding, large‐calibre metal stent covered with a biocompatible, biostable polymer, making it a non‐permeable tube that prevents tissue ingrowth into the lumen and early encrustation. The Allium stent offers longitudinal flexibility and radial force to maintain the patency of the ureter lumen and features a unique unravelling mechanism to facilitate its endoscopic removal.

### 1.3 Surgical technique

All procedures were performed by one of two experienced consultant endourologists in our centre. The procedure was conducted in the lithotomy position under general anaesthesia, with prophylactic antibiotics administered. Following 9.8‐F ureteroscopy, a guidewire was inserted to the renal pelvis under fluoroscopic guidance. In cases where the guidewire could not be passed, a smaller ureteroscope (7.5 or 4.5 F) was used for further exploration. The anterograde or retrograde ureterography was performed under X‐ray guidance to determine the location and length of the ureteral stenosis. A guidewire was reintroduced, and a ureteral balloon dilator, flexible ureteroscope sheath or ureteroscope was used to traverse the stenotic segment and perform dilation within 3 min. Finally, the Allium stent was placed into the stricture segment and confirmed by fluoroscopic imaging. Antibiotics and analgesics were routinely administered after surgery. Special cases with specific techniques are described below.

### 1.4 Statistical analysis

Data processing and analysis were performed using SPSS version 20 software (IBM, Armonk, NY, USA). The χ^2^ Pearson test was used to compare categorical variables. Independent variables were compared using Student's *t*‐test. Continuous variables were expressed as means with standard error of the mean (SEM), while categorical variables were presented as percentages. A *p*‐value of less than 0.05 was considered statistically significant.

## 2 RESULTS

As shown in Table 1, the study cohort consisted of 23 patients and 25 cases, with a mean age of 57.70 years (range 32–76 years). The primary sites of stricture included 11 cases in the left ureter, 10 in the right ureter, and two in the bilateral ureters. The mean length of the ureteral stricture was 4.5 cm (range 1–18 cm). The aetiology of ureteral stenosis was as follows: one patient (4.4%) due to failed UPJO pyeloplasty, two patients (8.7%) with a history of lithotripsy, six patients (26.1%) with iatrogenic injury or synechia from abdominal/pelvic surgery, seven patients (30.4%) due to tumour invasion, four patients (17.4%) who had received radiotherapy and left three patients (13.0%) due to other reasons. All stents were successfully inserted and correctly positioned in the 25 ureters. As summarized in Table 2, 12 cases (48%) were inserted Allium stent with balloon dilation, three cases (12%) with ureteral sheath and 10 cases (40%) with rigid scope dilation only. Before discharge, three patients (13.0%) occurred fever of Clavien Dindo Grade I. Besides, four (17.4%), six (26.1%) and four patients (17.4%) experienced severe fever, pain and haematuria of Clavien Dindo Grade II. As of December 2024, the long‐term stent patency rate was 68%, with a median follow‐up time of 39.5 months (range 13–67 months). For the eight cases of stent failure, the mean indwelling time of the Allium stent was 14 months, with the shortest recorded time being 2 months. The reasons for failure were as follows: stent migration in four patients (50%), stent occlusion due to encrustation in one patient (12.5%), persistent stenosis in two patients (25%) and severe infection in one patient (12.5%). Following stent failure, two patients (25%) had the Allium stent removed; two patients (25%) underwent robot‐assisted pyeloureteroplasty; four patients (50%) underwent ureteral lithotripsy, switch or replacement with a new Allium stent, or were switched to a different type of metal stent, respectively.

**TABLE 1:** Patients' characteristics and preoperative findings.

	Results
Total number of the patients, *n*	23
Total number of affected ureters, *n*	25
Gender, *n* (%)	
Male	10 (43.5%)
Female	13 (56.5%)
Age (year), median (range)	57.70 (32–76)
BMI (kg/m^2^), mean (range)	22.75 (16.96–27.73)
Serum creatinine (μmol/L), mean (range)	100.48 (35–275)
Haemoglobin (g/L), mean (range)	109.09 (70–145)
Affected side, *n* (%)	
Left	11 (47.8%)
Right	10 (43.5%)
Bilateral sides	2 (8.7%)
Length of ureteral stricture (cm), mean (range)	4.5 (1–18)
Location of ureteral stricture, *n* (%)	
Upper (including UPJ)	7 (28%)
Upper to middle	3 (12%)
Middle	1 (4%)
Middle to lower	4 (16%)
Lower	10 (40%)
Combined with abdominal/pelvic tumour, n (%)	
Yes	17 (73.9%)
No	6 (26.1%)
History of abdominal/pelvic surgery, *n* (%)	
Yes	17 (73.9%)
No	6 (26.1%)
History of abdominal/pelvic radiotherapy, *n* (%)	
Yes	9 (39.1%)
No	14 (60.9%)
Aetiology associated with ureteral stricture, *n* (%)	
Failed pyeloplasty for UPJO	1 (4.4%)
Lithotripsy	2 (8.7%)
Iatrogenic injury/synechia by abdominal/pelvic surgery	6 (26.1%)
Tumour invasion	7 (30.4%)
Radiotherapy	4 (17.4%)
Others	3 (13.0%)
Urine culture before stent, *n* (%)	
Positive	6 (26.1%)
Negative	17 (73.9%)

**TABLE 2:** Intraoperative details and follow‐up results.

	Results
Surgical procedure, *n* (%)	
Left Allium stent replacement	11 (47.8%)
Right Allium stent replacement	10 (43.5%)
Bilateral Allium stent replacement	2 (8.7%)
Dilation procedure	
Balloon, *n* (%)	12 (48%)
Flexible sheath, *n* (%)	3 (12%)
Ureteroscope, *n* (%)	10 (40%)
Operative time (min), mean (range)	108.39 (20–310)
Immediate stent patency, *n* (%)	25 (100%)
Long‐dated stent patency, *n* (%)	17 (68%)
Clavien Dindo Grade I before discharge, *n* (%)	
Fever	3 (13.0%)
Pain	0
Clavien Dindo Grade II before discharge, *n* (%)	
Infection requiring superior antibiotics or febrifuge	4 (17.4%)
Persistent pain requiring extra medicine	6 (26.1%)
Severe haematuria requiring medicine or transfusion	4 (17.4%)
LUTS requiring medicine	0
Clavien Dindo Grades III–IV before discharge, *n* (%)	0
Reasons of stent patency failure, *n* (%)	
Migration	4 (50%)
Upward	4
Downward	0
Encrustation	1 (12.5%)
Stenosis	2 (25%)
Severe infection	1 (12.5%)
Indwell time of stent patency failure case (month), mean (range)	14 (2–39)
Indwell time of stent patency success case (month), mean (range)	39.5 (13–67)
Final intervention for stent patency failure, *n* (%)	
Remove Allium stent	2 (25%)
Remove Allium stent and robot‐assisted pyeloureteroplasty	2 (25%)
Ureterolithotripsy	1 (12.5%)
Switch new Allium stent	1 (12.5%)
Add new Allium stent	1 (12.5%)
Switch new another kind of metal stent	1 (12.5%)

### 2.1 Special case presentation 1: The ruptured ureter was bridged with Allium stent by simultaneous percutaneous nephroscopy and ureteroscopy

**FIGURE 1:**
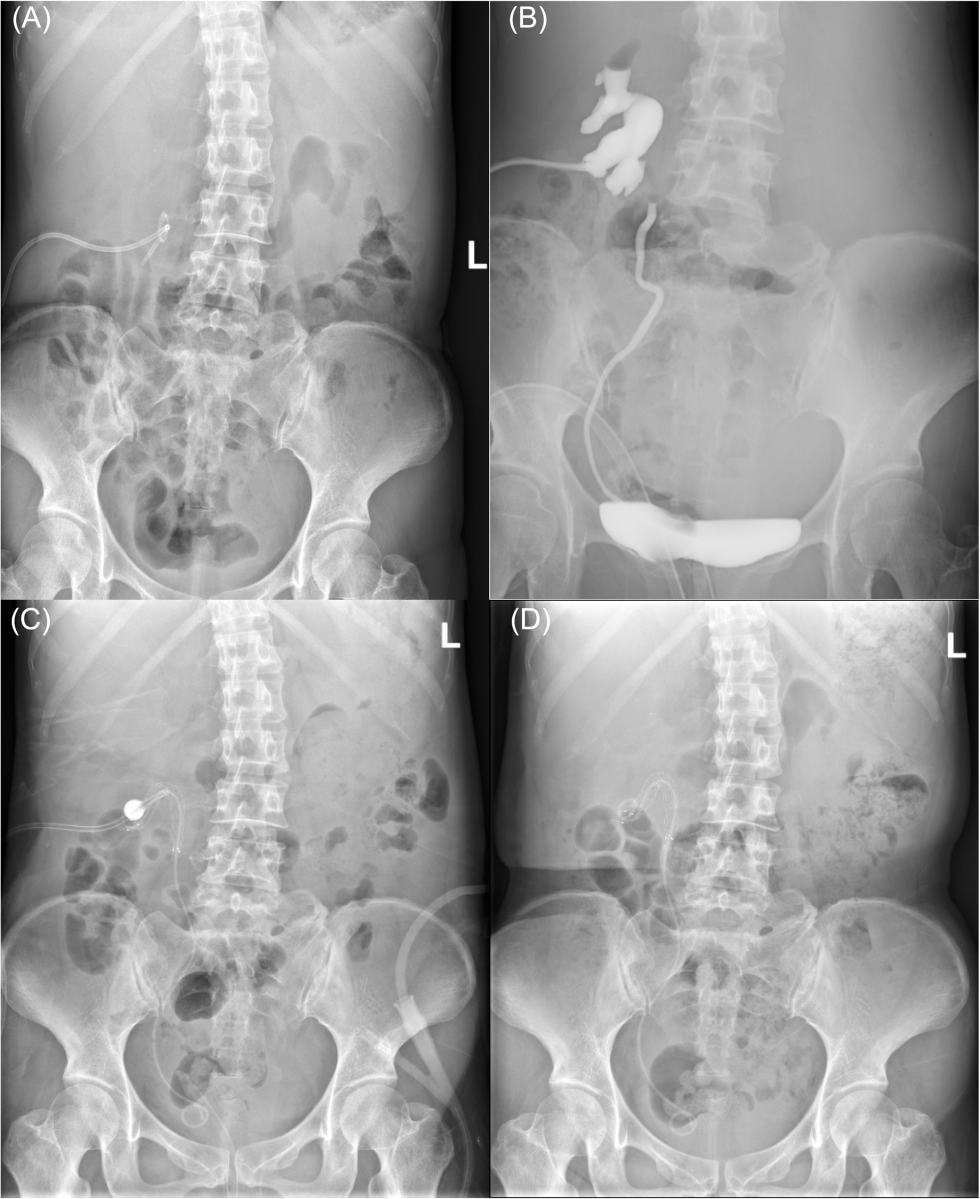
Imagings of the patient with right ruptured ureter before and after Allium stent insertion. (A) kidney, ureter and bladder (KUB) radiography showing the broken double‐J stent in right kidney, nephrostomy tube for urine drainage; (B) Antegrade and retrograde pyelography demonstrating the defect of contrast medium in right‐upper ureter; (C) KUB radiography showing the exact position of Allium stent bridging right ruptured ureter 2 weeks after surgery; (D) KUB demonstrating the exact position of Allium stent bridging right ruptured ureter 3 weeks after surgery without nephrostomy tube.

A 42‐year‐old Chinese woman with abdominal liposarcoma had undergone bilateral stent insertion, liposarcoma resection, right hemicolectomy, gallbladder resection and ileal transcolon anastomosis 3 months prior. After removal of the stent, part of the right double‐J stent was found broken in the renal pelvis, which was suspected to be a consequence of the previous surgery (Figure 1A–B). There was no evidence of renal function impairment, with a normal serum creatinine level. CT imaging revealed: (1) multiple patchy, water‐density foci extending from the surgical area of the right abdomen to the anterior pelvic cavity and the rectum; (2) a mass in the abdominal cavity that was slightly enlarged compared to previous imaging, measuring approximately 140 × 95 mm, with the residual lesion primarily located behind the liver, adjacent to the abdominal aorta, and with poorly defined boundaries with surrounding structures; and (3) a blurred periphery around the right renal pelvis, with foreign bodies visible within the renal pelvis (Video S1).

### 2.2 Surgical technique

After anaesthesia, the patient was placed in the oblique‐lithotomy position, and the procedure was performed by two urologists. The previous right nephrostomy tube was expanded to form F18 nephrostomy tract. Through this tract, the broken double‐J stent was removed, and the obstructed site in the renal pelvis was clearly visualized. The ureteroscope was then retrogradely introduced into the right ureter under the guidance of a guidewire, with the blind end of the ureter identified at its upper portion. After disrupting the blind end, the ureteroscope was advanced into the retroperitoneal space. Simultaneously, a nephroscope was used to traverse the obstructed site in the renal pelvis and enter the retroperitoneal space, where the ureteroscope with guidewire was clearly visible. The guidewire was then pulled out through the nephrostomy tract using the nephroscope. At this point, the ruptured ureter was bridged by the guidewire. A balloon dilator catheter was then placed over the guidewire to dilate the ruptured end of the ureter under X‐ray guidance. Finally, the Allium stent was introduced into the ruptured segment and released to bridge the tear in the ureter (Figure 1C–D). Six months post‐surgery, CT confirmed the normal position of the Allium stent (Video S2).

### 2.3 Special case presentation 2: Repairation of uretero‐vaginal fistula by Allium stent after comprehensive treatment to cervical cancer

A 64‐year‐old Chinese woman had undergone extensive laparoscopic hysterectomy, bilateral adnexectomy, pelvic lymph node dissection, bilateral ureteral stent implantation and cisplatin peritoneal irrigation 2 months prior. One month ago, she developed vaginal discharge but did not experience fever, abdominal pain or haematuria. Ureteroscopy at another hospital revealed a ‘left lower ureteral fistula’, and CT imaging showed a ‘left lower ureteral‐vaginal stump fistula and right hydronephrosis’ (Video S3). Pelvic MRI findings included as follows: (1) left ureter‐vaginal fistula; (2) multiple enlarged lymph nodes in the bilateral inguinal region, bilateral common iliac vessels and left internal iliac vessels, with metastatic tumours to be excluded; and (3) multiple cystic foci near the left external iliac blood vessel, with a pseudo‐encapsulated effusion. For further treatment of the ureteral‐vaginal fistula, the patient underwent ureteroscopic Allium stent placement under X‐ray guidance (Video S4).

### 2.4 Surgical technique

**FIGURE 2:**
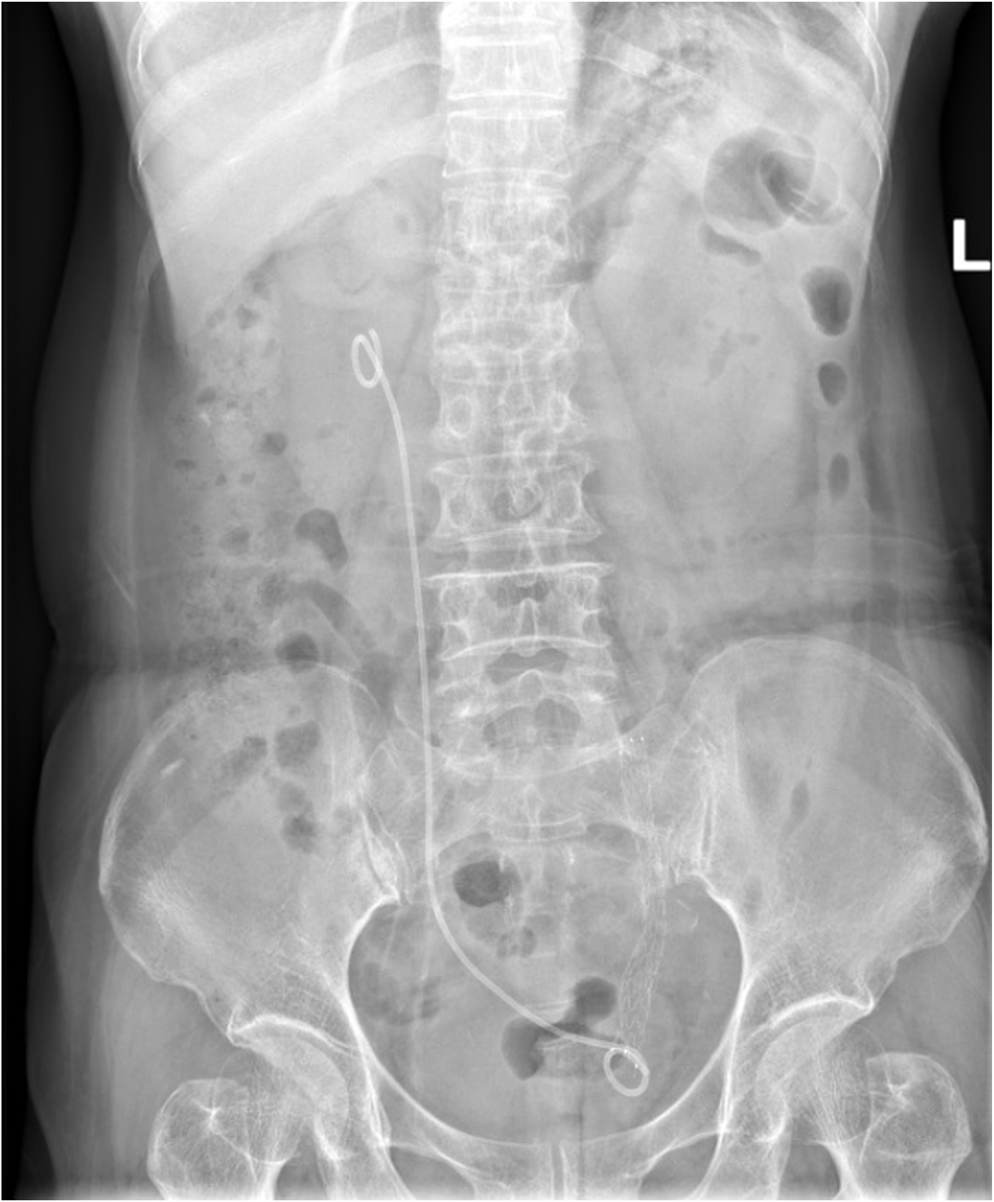
Kidney, ureter and bladder (KUB) radiography of Allium stent placement for repairing uretero‐vaginal fistula.

Ureteroscopy revealed a clear urethra with both ureteral orifices visible. A 9.8‐F ureteroscope was introduced into the left ureteral orifice under the guidance of a guidewire. Upon advancing 3 cm, a narrowed and occluded segment of the ureter with pale, stiff walls was identified. The guidewire was successfully passed through the narrowed segment, after which the ureteroscope was used to dilate this portion. The narrowed segment was approximately 3 cm in length as measured endoscopically. Next, methylene blue was injected through the former left nephrostomy tube and was able to pass smoothly through the entire ureter. A 12/14‐F flexible sheath was then introduced via the guidewire to further dilate the narrowed segment. After withdrawing the sheath, another guidewire was placed. An Allium stent was positioned, adjusted and released under X‐ray guidance along the guidewire. The right double‐J stent was removed, and a new 6‐F double‐J stent was placed under the guidance of the guidewire (Figure 2).

## 3 DISCUSSION

Allium stents represent a novel type of ureteral stents that can be used for any part of ureteral stenosis. In 2012, Moskovitz et al.^15^ first reported that the Allium stent provides an attractive and effective solution for long‐term internal ureteral drainage. Unlike traditional ureteral stents, Allium stents have unique characteristics, including a wide diameter, a polymer cover that prevents tissue ingrowth, the ability to anchor within the ureter and an increased radial force that helps keep the lumen open, even in the presence of significant external pressure.^19^ As a result, Allium stents can be used to treat tumours that compress or invade the ureter,^21^ urinary tuberculosis and radiation‐induced ureteral stenosis.^10^ The surfaces of Allium stents are coated with hydrophobic silane, and their copolymer‐coated structure (polyurethane) effectively reduces scaling and prevents tissue embedding and secondary infections.^15^, ^16^ Additionally, Allium stents provide local support that avoids complications, such as haematuria and irritation, often caused by the friction of double‐J stents on mucosal membranes.^15^, ^16^ Due to these features, Allium stents have been gradually adopted for the treatment of ureteral stenosis, showing remarkable results.^16^, ^17^, ^18^, ^19^, ^20^

A study by Bahouth et al.^16^ reported that patients with ureteral stenosis and long‐term indwelling Allium stents achieved clinical cure after stent removal. Wang et al.^22^ found that the stent patency rate was 84% during a median observation period of 16 months. Zhong et al.^23^ reported a success rate of 87.5% when using Allium stents to treat ureteral stricture after kidney transplantation. Moskovitz et al.^15^ reported a total success rate of 95% with Allium stents, with a median indwelling time of 17 months, although stent exchange and secondary luminal patency were also considered successful outcomes. In our study, Allium stents were successfully inserted in all patients, regardless of the severity or location of the stricture. Secondary stent exchanges, removal due to migration or adjustments for further relief of hydronephrosis were regarded as Allium stents failures. Consequently, our study's long‐term stent patency rate was 68%, with a median follow‐up time of 39.5 months (range 13–67 months). The differing success definitions may explain the lower stent patency rate observed in our study.

Theoretically, Allium stents are designed with anchor ends that prevent migration, thereby ensuring long‐term retention and reducing the need for replacement. Additionally, the material of Allium stents—nickel‐titanium memory alloy—has self‐expansion capabilities, which provides excellent support to the ureteral wall, further preventing stent migration. Despite these advantages, patients still experience stent shifts and detachments. Wang et al.^22^ reported stent migration in four out of 36 (12.5%) Allium stent placements. Moskovitz et al.^15^ reported after 49 Allium stents were implanted, seven were displaced (14.3%). Bahouth et al.^16^ reported that in 92 patients (107 sides) with ureteral stenosis, the incidence of stent displacement was 10.3% (11/107). Guandalino et al.^24^ found that 37% of stents were removed due to migration. Wang et al.^22^ speculated that the displacement of stents might be related to ureteral peristalsis and patient physical activity. An increase in human activity over time, along with prolonged stent implantation, may also contribute to stent translocation.^25^ In vitro studies have confirmed that blockage is more likely to occur when the end of the stent is exposed to the urine‐filled renal pelvis or bladder.^26^ Lu et al.^27^ speculated that displacement of Allium stent could be associated with the complete dilation of ureteral stenosis. However, in our study, all four patients who experienced stent migration had incomplete dilation of the stricture using a rigid ureteroscope, and the ends of their stents were exposed to the urine‐filled renal pelvis. Here, we propose a new possibility of Allium stents migration that when one end of the stent is released with little dilation and the other end is fully released, the stent status may be more susceptible to movement due to patient physical activity and remaining normal ureteral peristalsis.

Regarding special cases, Ditz et al.^28^ reported a case of a 46‐year‐old man with an incomplete tear of the proximal right ureter, who was treated with an Allium stent for definitive delayed repair. This demonstrated that Allium stent is an appropriate and safe option for managing ureteral injuries resulting from penetrating trauma. In our study, we performed simultaneous percutaneous nephroscopy and ureteroscopy to bridge a completely ruptured ureter with an Allium stent, further suggesting that Allium stent is a feasible option for severe ureteral injuries in patients who are not candidates for open or laparoscopic surgery. Additionally, we present a case of a 64‐year‐old woman with a uretero‐vaginal fistula. Due to her history of laparoscopic surgery, we chose an endoscopic approach and repaired the fistula using the Allium stent. In summary, these two cases highlight that Allium stent can be a minimally invasive procedure for managing special ureteral injuries, offering a viable alternative for patients who may not be suitable for more invasive surgical options.

This study has several limitations, including the small sample size and its retrospective design. Besides, our study presented all successful procedure cases, but in real life, not all the procedures of stent insertion succeed in 100%. According to our experience, it is not recommended Allium stent placement for patients who have the opportunity for laparoscopic or endoscopic surgery, such as patients with no history of surgical intervention, radiation therapy, multiple abdominal surgeries or cannot afford Allium stents. In conclusion, Allium stents are effective in treating ureteral stenosis to a certain extent. However, complications such as stent displacement can increase the complexity of subsequent surgical procedures. Therefore, the suitability of Allium stents should be carefully evaluated in patients with ureteral stenosis, and it should be considered as an option primarily for patients who are unfit for more invasive treatments but could potentially benefit from its use.

## AUTHOR CONTRIBUTIONS

Hanqi Lei contributed to manuscript writing, experiments and data analysis. Yajiao Cui contributed to experiments and data analysis. Mengjun Huang contributed to manuscript writing and essay polishing. Yamei Li and Donggen Jiang contributed to manuscript design. Jun Pang contributed to manuscript design and writing and manuscript writing.

## CONFLICT OF INTEREST STATEMENT

The authors declare that they have no competing interests.

## Supporting information


**Video S1.** Supporting Information.

**Video S2.** Supporting Information.

**Video S3.** Supporting Information.

**Video S4.** Supporting Information.

## Data Availability

All data generated or analysed during this study are included in this published article.
